# Voriconazole therapeutic drug monitoring: retrospective cohort study of the relationship to clinical outcomes and adverse events

**DOI:** 10.1186/1471-2334-13-105

**Published:** 2013-02-26

**Authors:** Helen Y Chu, Rupali Jain, Hu Xie, Paul Pottinger, David N Fredricks

**Affiliations:** 1 Division of Allergy & Infectious Diseases, Department of Medicine, University of Washington, Seattle, WA, USA; 2 Department of Pharmacy, University of Washington Medical Center, Seattle, WA, USA; 3 Vaccine and Infectious Diseases Division, Fred Hutchinson Cancer Research Center, Seattle, WA, USA

**Keywords:** Voriconazole, Therapeutic drug monitoring, Antifungals, Clinical efficacy

## Abstract

**Background:**

Voriconazole is approved for treatment of invasive aspergillosis and other invasive fungal infections, but the role for therapeutic drug monitoring (TDM) is not clear.

**Methods:**

We performed a retrospective cohort study of patients at the University of Washington Medical Center and Fred Hutchinson Cancer Research Center from 2007–2009. We compared the effect of therapeutic levels on clinical outcomes and evaluated the relationship between drug levels and adverse events.

**Results:**

A total of 108 patients had voriconazole TDM performed, of whom 84 (77.8%) had a hematologic malignancy and 47 (43.5%) had undergone hematopoietic stem cell transplantation. The primary reasons for treatment were presumed pulmonary aspergillosis (n = 83, 76.8%), other invasive mould infections (n = 13, 12.0%) and candidiasis (n = 9, 8.3%). There was a high degree of variability in voriconazole drug levels among patients (r^2^ = 0.01; range, <0.10 - 20 mg/L). Of the 46 patients with proven or probable invasive fungal disease, 25 (54.3%) achieved partial or complete response to therapy. There was no significant relationship between therapeutic drug levels and achievement of complete or partial response at 12 weeks (OR 0.29, 95% CI: 0.05-1.34) or radiologic response (OR 1.46, 95% CI: 0.32-7.83). Overall, 45 (41.7%) patients experienced adverse events. Voriconazole levels > 5.5 mg/L were not associated with increased incidence of encephalopathy (OR 3.08, 95% CI 0.79-11.0) or hepatotoxicity (OR 2.45, 95% CI 0.49-10.1).

**Conclusions:**

Voriconazole therapeutic drug levels were not associated with improvement in clinical outcomes among patients with proven or probable invasive fungal disease. We also did not find an association between supratherapeutic drug levels and hepatoxicity or encephalopathy. It is possible that the utility of voriconazole therapeutic drug monitoring to improve clinical efficacy or decrease adverse events may be limited to a subset of high-risk patients.

## Background

Invasive fungal infections (IFIs) are associated with high morbidity and mortality, particularly among immunocompromised patients. Voriconazole therapy is used for treatment of IFIs and is the drug of choice for treatment of invasive aspergillosis [1-3], although it is associated with hepatotoxicity and central nervous system adverse effects [4,5]. Drug levels are highly variable due to nonlinear pharmacokinetics and depend on many additional factors including patient age, presence of genetic polymorphisms that affect drug metabolism, and use of concomitant medications [6-9]. Prior observational studies suggest that monitoring of voriconazole drug levels predicts response, decreases adverse events among patients with invasive fungal infections, and may improve outcomes [7,10-13]. Specifically, they have found a role for a therapeutic concentration range of 1.0 to 5.5 mg/L to maximize therapeutic efficacy and minimize adverse events [10]. However, other factors also influence clinical outcomes, including immune status of the host, patient age, surgical resection of infected tissue, and co-morbidities [14-19]. A recent randomized controlled trial at a single center in Korea comparing the use of routine TDM with non-TDM for invasive fungal infections showed no difference in adverse events with and without drug monitoring [20]. The investigators concluded that among those patients in whom TDM was performed there was a significant reduction in discontinuation of antifungal therapy and improvement in achievement of complete or partial clinical response. Expert guidelines do not contain specific recommendations for therapeutic drug monitoring although they suggest that plasma drug levels may be useful in cases of therapeutic failure or potential drug toxicity [1]. We performed a retrospective cohort study of patients at our institution who had therapeutic drug monitoring (TDM) of their voriconazole therapy. We hypothesized that therapeutic drug levels were associated with improved clinical outcomes, and supratherapeutic levels would be associated with adverse events, in particular neurotoxicity and hepatoxicity in our patient population.

## Materials and methods

### Ethics statement

This protocol was approved by the University of Washington School of Medicine Institutional Review Board. Written consent was given by the patients for their information to be stored in the hospital database and used for research.

### Patients

Using pharmacy records, we identified all consecutive inpatients and outpatients at the University of Washington Medical Center, Harborview Medical Center, and the Seattle Cancer Care Alliance (all in Seattle, Washington, USA) who were treated with intravenous or oral voriconazole therapy and had drug level monitoring performed between July 1, 2007 and July 31, 2009. We excluded patients under 18 years of age and those on voriconazole therapy who did not have TDM performed.

### Methods

We performed electronic chart review of all patients included in our study. The European Organization for Research and Treatment of Cancer and Mycoses Study Group (EORTC/MSG) guidelines were used for classification of proven, probable, or possible invasive fungal infections [21]. In all patients, baseline sociodemographic data, clinical characteristics, presence of adverse events, clinical outcomes, timing, dose, and method of administration of voriconazole (intravenous or oral) and frequency and timing of TDM were recorded. Voriconazole drug levels were measured using validated high-pressure liquid chromatography tandem mass spectrometry assays at the Mayo Clinic (Mayo Medical Laboratories, Rochester, MN) [22]. We defined subtherapeutic levels as < 1.0 mg/L, therapeutic levels as 1.0-5.5 mg/L, and supratherapeutic levels as > 5.5 mg/L [10]. Clinical outcomes were assessed using clinical, radiologic, and microbiologic criteria at 6 and 12 weeks from start of therapy and categorized as complete response to therapy, partial response to therapy, progression of disease or no response to therapy. This final category included those who died, did not respond to therapy, or had cessation of therapy due to adverse drug events. Neutropenia was defined as an absolute neutrophil count (ANC) less than 1500/mm^3^. We defined hepatoxocity as AST/ALT >5x upper limit of normal or alkaline phosphatase/total bilirubin > 3× upper limit of normal [10]. We defined renal failure as a creatinine increase of 0.3 mg/dL in 48 hours [10]. We defined use of combination antifungals as administration of micafungin, caspofungin, or amphotericin B in conjunction with voriconazole.

Data were recorded using Project Redcap and analyzed using Stata 11.0 (StataCorp, College Station, TX) and SAS version 9.1 (SAS Institute, Inc., Cary, NC) [23]. Correlation coefficients were calculated for the relationship of drug level to initial voriconazole dose. Median of first, second, and third drug levels were compared using the Kruskal-Wallis test. Fisher’s exact, X^2^ tests, and t-tests with unequal variance were used to compare dosing characteristics and response to therapy in patients with drug levels < 1.0 mg/L versus ≥1.0 mg/L and to compare adverse drug events in patients with drug levels ≤5.5 mg/L versus >5.5 mg/L. Among patients with proven or probable disease, the probabilities of therapeutic outcomes were summarized using cumulative incidence estimates and compared using the log-rank test where death or progression to disease were considered as competing risks among patients with and without therapeutic drug levels (<1.0 mg/L versus ≥1.0 mg/L). The presence of baseline confounders was compared in these two groups using t-tests with unequal variance.

## Results

Altogether, 108 patients had voriconazole TDM performed (Table 1) between 2007 and 2009. Fifty-nine (54.6%) of patients were male. Eighty-five (78.7%) were white, seven (6.5%) were Asian, and five (4.6%) were African-American. Seventy-six patients (70%) were inpatient at the University of Washington and 29 were outpatients (27.1%). Eighty-four (77.8%) patients had a hematologic malignancy, of whom 47 (55.9%) had undergone hematopoietic cell transplantation. Of the transplants performed, 21 (43.8%) were matched unrelated allogeneic stem cell transplants, 14 (29.2%) were matched related allogeneic stem cell transplants, seven (14.6%) were mismatched unrelated allogeneic stem cell transplants, and six (12.5%) were autologous stem cell transplants. Twenty-eight patients had a diagnosis of graft versus host disease. Among patients who were neutropenic at the start of voriconazole therapy (n = 37), the median duration of neutropenia was 37 days [18, 56]. The primary reasons for treatment were suspected or confirmed pulmonary aspergillosis (n = 83, 76.8%), other invasive fungal infections (n = 13, 12.0%), candidiasis (n = 9, 8.3%) and febrile neutropenia (n = 9, 8.3%). Other fungal organisms included *Fusarium* (n = 2), *Absidia* (n = 1), *Alternaria* (n = 1), *Saccharomyces* (n = 1), *Scedosporium* (n = 1), *Ascomyctes* (n = 1), *Rhizopus* (n = 1), *Pseudoallescheria* (n = 1), and *Paecilomyces* (n = 1).

**Table 1:** Sociodemographic and clinical characteristics of patients receiving voriconazole TDM between 2007-2009

***Variable***	***Patients with voriconazole drug monitoring [n, (%)]***
Number of patients	108
Median Age (IQR)	53 (38–64)
Sex, % male	59 (54.6%)
**Underlying condition**	
Hematopoietic stem cell transplant	47 (43.5%)
Hematologic malignancy without a stem cell transplant	37 (34.3%)
Solid organ transplantation	10 (9.3%)
Other condition	9 (8.3%)
None	3 (2.8%)
Reason for voriconazole treatment*	
Invasive pulmonary aspergillosis	83 (76.8%)
Invasive sinus or CNS aspergillosis	5 (4.6%)
Invasive candidal infection	9 (8.3%)
Other invasive fungal infection^δ^	13 (12.0%)
Febrile neutropenia	9 (8.3%)
**Diagnosis of infection**	
Proven or probable invasive fungal disease	46 (42.5%)
Possible invasive fungal disease	43 (39.8%)
**Voriconazole initial drug level**	
Subtherapeutic (<1.0 mg/L)	32 (29.6%)
Therapeutic (1.0-5.5 mg/L)	64 (59.2%)
Supratherapeutic (>5.5 mg/L)	12 (11.1%)

*Sixteen patients received voriconazole therapy for more than one indication.

^δ^ Other fungal organisms included *Fusarium* (n = 2), *Absidia* (n = 1), *Alternaria* (n = 1), *Saccharomyces* (n = 1), *Scedosporium* (n = 1), *Ascomyctes* (n = 1), *Rhizopus* (n = 1), *Pseudoallescheria* (n = 1), and *Paecilomyces* (n = 1).

Twenty-one (19.4%) patients received concomitant tacrolimus therapy, 47 (43.5%) received proton-pump inhibitors, and nine (8.3%) received additional antifungal therapy with micafungin, caspofungin, and/or amphotericin B. The median weight at start of voriconazole therapy was 71.3 kg (range, 60.5-85.8 kg).

**Figure 1:**
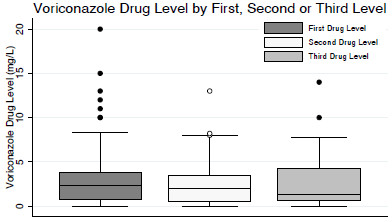
**Comparison of median voriconazole drug levels.** Median voriconazole drug level did not vary by first, second or third drug level among patients who received serial drug level monitoring (*P* = 0.55).

The median duration of voriconazole therapy (n = 85) was 35 days (range, 13–92 days) (Table 2). All patients had at least one drug level checked after initiation of therapy, 51 patients had at least two drug levels, and 26 patients had at least three drug levels. There was no significant difference between median initial level (2.4 mg/L; range, 0.7 - 3.8 mg/L), second level (2.0 mg/L; range, 0.5 - 3.5 mg/L), or third level (1.4 mg/L; range, 0.6 - 4.2 mg/L) (*P* = 0.55, Figure 1). In patients in whom the date of voriconazole initiation was recorded (n = 100), the median time between start of voriconazole therapy and initial drug level was 11 days (range, 3–164 days), with the first drug level checked at least two days after initiation of therapy. Among the 64 patients for whom data were available, we found that the median time between voriconazole dose administration and drug level testing was 11.3 hours (range, 8.9-12.0 hours) and that 31% of levels were checked 10–12 hours after the last voriconazole dose.

**Table 2:** Voriconazole dosing characteristics, drug level monitoring, and duration of therapy

***Voriconazole therapy***	***Median value[(IQR]***
Voriconazole serial drug levels (mg/L)	
Initial drug level (n = 108)	2.35 [0.7, 3.8]
Second drug level (n = 51)	2.00 [0.5, 3.5]
Third drug level (n = 26)	1.35 [0.6, 7.7]
Voriconazole loading dose (mg/dose; n = 59)	400 [350, 480]
Voriconazole maintenance dose (mg/dose; n = 107)	260 [200,300]
Patient weight (kg; n = 107)	71.3 [60.5, 85.8]
Duration of voriconazole therapy in days (n = 85)	35 [13, 92]
Days between initiation of voriconazole to first drug level (n = 100)	11 [3,164]
Hours between last dose and trough drug level (n = 64)	11.3 [8.9-12.0]

**Figure 2:**
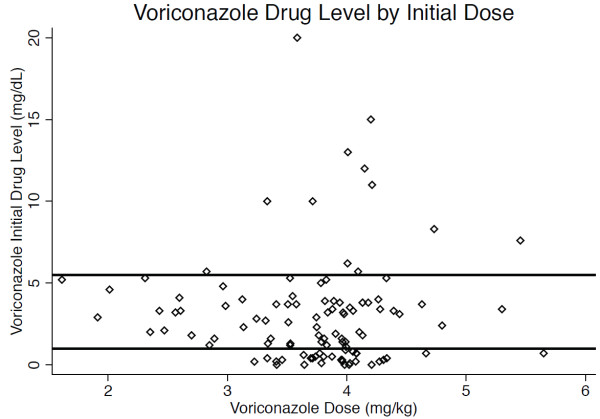
**Comparison of voriconazole initial drug level by weight-adjusted dosage received.** Initial drug level did not correlate with weight-adjusted dosage of voriconazole (r^2^ = 0.01; n = 107). Area between the horizontal lines indicates the therapeutic range between 1.0 and 5.5 mg/L.

There was no correlation between initial drug level and weight-adjusted voriconazole dosage (r^2^ = 0.01; Figure 2). When a range of 1.0-5.5 mg/L was considered therapeutic, 32 patients (29.6%) had subtherapeutic initial drug levels, 64 (59.3%) had therapeutic drug levels, and 12 (11.1%) had supratherapeutic levels. Among patients with subtherapeutic initial drug levels, 20 had a second level checked, and 10 (50%) of the second levels remained subtherapeutic. Among patients with supratherapeutic initial drug levels, five had a second drug level drug checked, and four (80%) remained supratherapeutic.

**Figure 3:**
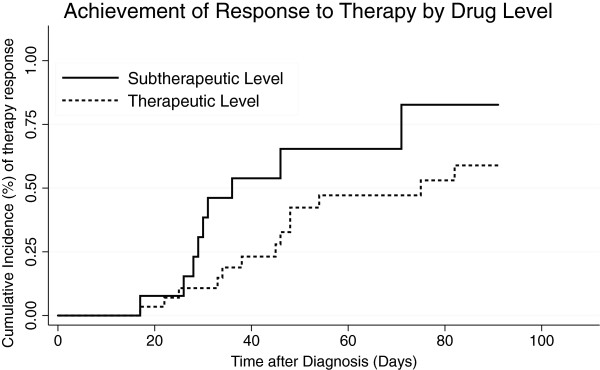
**Cumulative incidence graph comparing therapy response with therapeutic versus subtherapeutic initial drug levels shows no statistically significant difference in complete or partial response to therapy at 12 weeks among a subset of patients with proven or probable invasive fungal disease (n = 46; HR: 0.45; *P*** **= 0.07).**

Eight (7.4%) patients had proven invasive fungal disease, 38 (35.1%) had probable disease, and 43 (39.8%) had possible disease. Overall, 43 (39.8%) patients achieved partial or complete response to therapy at 12 weeks, while 18 (16.7%) had invasive fungal disease progression. In the subset of patients with proven or probable invasive fungal disease (n = 46), 22 (47.8%) achieved partial or complete response to therapy at 12 weeks and 11 (23.9%) had disease progression (Table 3). Among patients with proven and probable disease, there was no significant relationship between therapeutic drug levels and achievement of complete or partial response at 12 weeks (Figure 3; OR 0.29, 95% CI: 0.05-1.34) although we did observe a significant relationship between subtherapeutic initial drug levels and achievement of complete or partial response at 6 weeks (OR 0.19, 95% CI 0.04-0.96). Among those with pulmonary aspergillosis, radiologic response, as defined by decrease in size of pulmonary nodules on CT scan, was noted in 37/67 (55.2%). There was no significant relationship between therapeutic drug levels and radiologic response (OR 1.46, 95% CI: 0.32-7.83). We further examined the subset of patients who had complete or partial response at 6 weeks with initial subtherapeutic drug levels. Of these, all seven had invasive pulmonary aspergillosis. Three were stem cell transplant recipients, two had received lung transplants, one had chronic obstructive pulmonary disease, and one patient had acquired immunodeficiency syndrome. The median time between start of voriconazole therapy and monitoring was seven days (range, 4–26 days). Of the four patients who had a repeat level checked, two remained subtherapeutic and two had a therapeutic level.

**Table 3:** The relationship between voriconazole initial drug levels and clinical and radiologic response to therapy at 6 and 12 weeks of follow-up

***Variable***	***Voriconazole level < 1.0 mg/L***	***Voriconazole level ≥1.0 mg/L***	***OR [95% CI]***	***p-value***
Intravenous maintenance dose administration	11 (35.4%)	26 (36.1%)	1.02 [0.39-2.77]	0.95^†^
Voriconazole median dosage (mg)	280 (170–400)	255 (125–480)	--	0.61^‡^
Response to antifungal therapy at 6 weeks*				
Complete or partial response (n = 13)	7/13 (53.8%)	6/33 (18.2%)	0.19 [0.04-0.96]	0.03 ^§^
Progression of disease (n = 9)	1/13 (7.7%)	8/33 (24.2%)	3.84 [0.42-184.3]	0.41 ^§^
Response to antifungal therapy at 12 weeks*				
Complete or partial response (n = 22)	9/13 (69.2%)	13/33 (39.4%)	0.29 [0.05-1.34]	0.10 ^§^
Progression of disease (n = 11)	3/13 (23.1%)	8/33 (24.2%)	1.07 [0.20-7.50]	1.00 ^§^
Radiologic improvement*				
Pulmonary nodule resolution or decrease in size at 6 weeks (n = 46)	4 (30.8%)	13 (39.4%)	1.46 [0.32-7.83]	0.74 ^§^

* In patients with proven or probable invasive fungal disease (n = 46).

† Chi-squared test.

‡ Two-sample t-test.

§ Fisher’s exact test.

We examined for the presence of confounders in patients with and without therapeutic drug levels, and found no statistically significant baseline differences in gender, steroid use, duration of voriconazole therapy, obesity (defined as weight ≥ 90 kg), duration of neutropenia ≥ 10 days, presence of graft-versus-host-disease, or use of combination antifungals.

**Figure 4:**
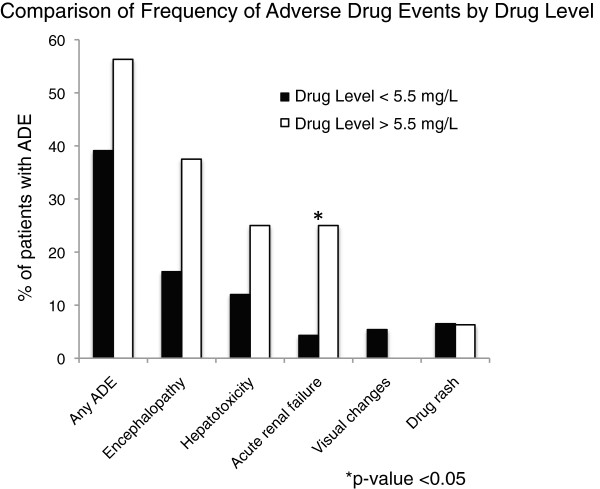
**Comparison of frequency of adverse drug events in patients with and without supratherapeutic drug levels, defined as a level > 5.5 mg/L.** The y-axis represents the percentage of patients with adverse drug events. The total number of adverse drug events, as well as individual drug events, are noted on the x-axis. There was no statistically significant difference in the frequency of overall adverse drug events, or of encephalopathy, hepatoxicity, skin rash, or visual changes among patients with and without supratherapeutic voriconazole drug levels. There was a statistically significant difference in incidence of acute renal failure (*) between the two groups.

Among all patients, we recorded adverse events in the time period within the first month of therapy or the duration of hospitalization, whichever was longer. We observed 45 (41.7%) adverse events with 21 cases of encephalopathy and 15 cases of hepatotoxicity (Table 4, Figure 4). Among the 15 cases of hepatotoxicity, only two received initial doses greater than 4 mg/kg. Voriconazole levels > 5.5 mg/L at any time during therapy (n = 16) were not associated with the overall occurrence of adverse events (OR 2.00; 95% CI 0.60-6.89). Specifically, there was no significant association between supratherapeutic levels and hepatotoxicity (OR 2.45; 95% CI: 0.49-10.0) or encephalopathy (OR 3.08; 95% CI: 0.79-11.0). There was significantly more acute renal failure in patients with supratherapeutic drug levels (OR 7.33; 95% CI 1.17-43.8). Patients with and without supratherapeutic drug levels did not differ in baseline creatinine prior to start of voriconazole or in use of the intravenous formulation of voriconazole. There were no significant differences in presence of visual changes or drug rash between the two groups.

**Table 4:** The relationship between supratherapeutic voriconazole drug levels during therapy and adverse drug events

***Variable***	***Voriconazole level ≤ 5.5 mg/L (*n = *92)***	***Voriconazole level > 5.5 mg/L (*n = *16)***	***OR [95% CI]***
Any adverse drug event*	36 (39.1%)	9 (56.3%)	2.00 [0.60-6.89]^*†*^
Adverse drug event by subtype*			
Encephalopathy	15 (16.3%)	6 (37.5%)	3.08 [0.79-11.0]^*†*^
LFT abnormalities (AST/ALT > 5x ULN, Alk phos/bili >3x ULN)	11 (12.0%)	4 (25.0%)	2.45 [0.49-10.1]^*†*^
Acute renal failure (creatinine rise ≥ 0.3 mg/L in 48 hours)^§^	4 (4.3%)	4 (25.0%)	7.33 [1.17-43.8]^*†*^
Visual changes	5 (5.4%)	0 (0%)	--
Drug rash	6 (6.5%)	1 (6.3%)	0.96 [0.02-8.81]^*†*^

*For at least one month after initiation of therapy.

†Fisher’s exact test.

^§^Baseline creatinine 1.1 in 4 subjects with therapeutic levels, and 1.3 in 4 subjects with supratherapeutic levels, all with eventual acute renal failure.

## Discussion

In our patient population, the majority of patients receiving drug monitoring had hematologic malignancy and presumptive invasive aspergillosis, although fewer than half (n = 46; 43%) had proven or probable invasive fungal disease. Consistent with prior studies, there was high variability in initial drug levels, and those levels did not correlate with voriconazole dosage [8,15,24,25]. The time between initiation of voriconazole and initial drug level was 11 days in our study. Despite the long duration between drug initiation and TDM, one-third of our patients had subtherapeutic initial drug levels. When we compared dosing regimens in patients with levels < 1.0 mg/L to those with levels ≥ 1.0 mg/L, we found no difference in the proportion who received intravenous formulations or weight-adjusted dosage, suggesting that weight and route of administration are not the primary determinants of drug levels [26,27].

Furthermore, similar to prior studies, we found that the majority of patients who had subtherapeutic or supratherapeutic levels on initial testing had similar subsequent results. Because of the nature of our study, we do not have data regarding the presence of genetic polymorphisms that affect drug metabolism such as CYP2C19 in our population that could explain some of the variability in initial drug levels [15,28,29]. A further analysis of clinical and radiologic outcomes among the patients who were Caucasian, in whom the CYP2C19 polymorphism is more prevalent, did not reveal any difference in associations between drug level and outcome as compared to the total study population. It is also possible that for a subset of our patients, voriconazole drug levels are not reflective of true trough concentrations because of inappropriate timing of drug level monitoring relative to voriconazole dose administration. This may be particularly true among outpatients for whom we do not have information regarding dosage in relationship to drug level monitoring. In prior studies, there has been high variability in physician ordering practices of voriconazole levels, including random drug levels being checked rather than true trough levels [30]. In our study, however, we found that the median time after dose administration was close to ideal at 11.3 hours. Nonadherence to therapy is also a factor that is associated with subtherapeutic drug levels, and has been examined in prior studies by measuring presence of voriconazole metabolites in patient blood samples [15]. These factors are less likely to be the case in our study given clear documentation of the timing of dose administration and blood sample collection among our inpatients, though this data is often incomplete for our outpatient population.

No significant relationship between achievement of therapeutic drug levels and clinical response to therapy at 12 weeks or radiologic response was demonstrated among patients with proven or probable invasive fungal infection. Surprisingly, at 6 weeks, there was a significant association between subtherapeutic drug levels and clinical response. These results are in contrast to prior studies, which do show a relationship between therapeutic drug levels and clinical efficacy [7,10,11]. This discrepancy may be a result of limiting our analysis of clinical outcomes to patients with proven or probable IFIs. Prior studies have examined clinical response in all patients with voriconazole TDM, including those without proven or probable IFI [10]. In addition, the timing of clinical outcome assessment may have been different from prior studies, as well as the sociodemographic profile of the patient population [31]. We chose to perform our assessment at 6 and 12 weeks, rather than at 2 weeks, due to the prolonged period of immunosuppression and duration of antifungal therapy in many of our patients.

An important potential selection bias in our study was the selective inclusion of patients for whom voriconazole TDM was performed. There were many patients who received voriconazole who did not have their levels monitored, and we were unable to capture data on these patients. The decision to order voriconazole levels was at the discretion of the clinician. An algorithm was put into place for routine ordering of voriconazole levels, though this was not implemented until early 2009, and did not affect most of the patients whom we studied. We have, however, attempted to address the bias by performing the evaluation of clinical outcome only among patients with proven and probable invasive fungal infection, for whom initial voriconazole TDM was likely to be performed regardless of response to therapy. However, selection bias could potentially explain why we did not show a relationship between achievement of therapeutic drug levels and clinical response.

We were limited in our study by an inability to capture mortality data, given the retrospective nature of the chart review and the loss to follow-up among some inpatients who transitioned to outpatient care. However, for the majority of patients, we were able to obtain outcomes measured at 6 and 12 weeks after initiation of voriconazole therapy, and we have based our analysis on these endpoints. We also do not have information on the microbiologic isolates of the pathogens to establish the relationship between voriconazole serum concentration and pathogen MICs, as performed in prior studies because this was not routinely performed at our institution in 2007–2009 [32,33]. We explored whether differences in confounders between patients with subtherapeutic and therapeutic levels could account for the difference in therapeutic outcome. We did not find differences between the two groups in weight, steroid use, incidence of graft-versus-host-disease, duration of therapy, or use of combination antifungal agents. We were unable to evaluate the effect of sepsis as a potential confounder because many of our patients had prolonged complicated hospitalizations related to their hematologic malignancies, and it was difficult to determine whether sepsis during their hospitalization had an effect on their voriconazole concentrations. We speculate that in our population with a high proportion of patients with hematologic malignancy and hematopoietic stem cell transplantation, host immune response is an important predictor of outcome.

There is currently no consensus on optimal timing for voriconazole drug monitoring. It is unclear whether routine voriconazole drug monitoring should be performed, or whether the clinical utility of TDM is limited to patients with adverse events. The study by Dolton et al. included all patients for whom trough levels were checked on day 2 of dosing in those with loading doses, and on day 7 or later in those without loading doses [34]. All of our patients had drug monitoring performed at least 3 days after initiation of therapy, suggesting that they had reached a steady-state level by time of monitoring.

A high frequency of adverse drug events was seen in our patient population, with a higher incidence of acute renal failure in patients with supratherapeutic levels. Despite prior reports associating cyclodextrin in intravenous formulations with renal failure [17], we did not find a difference in prevalence of intravenous dose administration between patients who did and did not have renal failure. We also found no association between supratherapeutic levels and hepatotoxicity, in contrast to prior studies which have noted significantly higher levels of liver function test abnormalities with higher trough levels [11,29]. Also, we did not find that the patients with hepatotoxicity received doses higher than the standard 4 mg/kg dosing, consistent with prior data showing that higher doses do not correspond to hepatoxicity [5]. Similar to prior studies, a trend suggesting that supratherapeutic voriconazole levels were associated with encephalopathy was seen, though this was not a statistically significant association [18]. It is possible that the nature of the chart review did not allow us to capture information regarding the presence of central nervous system effects because it relied on provider charting of symptoms. However, overall our population had rates of adverse events similar to prior studies suggesting that despite use of chart review for data extraction, we were able to capture a substantial proportion of the adverse events experienced by our patients [35].

Our results stand in contrast to a recent randomized controlled trial conducted at a single center in Korea showing improvement in clinical outcomes and decrease in discontinuation of therapy due to adverse events with therapeutic drug monitoring [20]. We believe that this reflects both a difference in our patient population and the absence of a clinical trial design in our study. Participation in clinical trials may change the behavior of physicians and patients, and results are often difficult to replicate in clinical practice [36]. Our study reflects the outcomes seen in practice at an academic center highly experienced with management of invasive fungal infections in immunocompromised hosts. We propose that further studies be performed to evaluate whether implementation of the clinical trial algorithm is efficacious in a clinical setting.

## Conclusions

In conclusion, we observed that in clinical practice, monitoring of voriconazole therapeutic drug levels did not have any effect on adverse events or on clinical outcomes in a population with a high rate of hematologic malignancy and invasive fungal infections. We suggest that further prospective studies should be performed to evaluate the relationship between drug levels and therapeutic efficacy at multiple centers. These studies should include an evaluation of adherence to TDM monitoring algorithms, and aim to identify the particular subset of patients for whom drug monitoring yields benefits in terms of clinical efficacy and avoidance of adverse drug events.

## Competing interests

All authors declare no conflicts of interest.

## Authors’ contributions

All authors contributed to the work presented in this paper. HC, RJ, PP, and DF jointly conceived the study. HC designed the study, gathered the data and performed statistical analysis with assistance from HX. HC wrote the manuscript with contributions from other authors. HX performed the statistical analysis. RJ, DF, and PP gave conceptual advice and technical support. All authors discussed the results and implications and commented on the manuscript at all stages. All authors read and approved the final manuscript.

## Pre-publication history

The pre-publication history for this paper can be accessed here:

http://www.biomedcentral.com/1471-2334/13/105/prepub
